# Pathologic complete response and outcomes by intrinsic subtypes in NSABP B-41, a randomized neoadjuvant trial of chemotherapy with trastuzumab, lapatinib, or the combination

**DOI:** 10.1007/s10549-019-05398-3

**Published:** 2019-08-19

**Authors:** Sandra M. Swain, Gong Tang, Peter C. Lucas, André Robidoux, David Goerlitz, Brent T. Harris, Hanna Bandos, Charles E. Geyer, Priya Rastogi, Eleftherios P. Mamounas, Norman Wolmark

**Affiliations:** 1 National Surgical Adjuvant Breast and Bowel Project (NSABP), Nova Tower 2, 100 Allegheny Square, Pittsburgh, PA 15212 USA; 2 Georgetown Lombardi Comprehensive Cancer Center, Georgetown University Medical Center, 4000 Reservoir Road NW, 120 Building D, Washington, DC 20057 USA; 3 University of Pittsburgh, 4200 Fifth Ave, Pittsburgh, PA 15260 USA; 4 Centre Hospitalier de L’Universite ´ de Montréal, 3840 St Urbain St, Montreal, QC H2W 1T8 Canada; 5 Massey Cancer Center, Virginia Commonwealth University, 401 College Street, Box 980037, Richmond, VA 23298 USA; 6 Magee-Womens Hospital, 300 Halket Street, Pittsburgh, PA 15213 USA; 7 Orlando Health UF Health Cancer Center, 1400 S Orange Avenue, Orlando, FL 32876 USA

**Keywords:** Breast cancer, HER2 enriched, Intrinsic subtype, Genomic, Neoadjuvant, Trastuzumab

## Abstract

**Purpose:**

NSABP B-41, a phase three randomized trial, evaluated neoadjuvant lapatinib, trastuzumab, or the combination with chemotherapy in patients with HER2-positive operable breast cancer. Though no significant difference in pathologic complete response (pCR) was found among the three arms, pCR was associated with prolonged survival. We analyzed tumor intrinsic subtypes with Prediction Analysis of Microarray 50 in a subset of B-41 patients to determine their value in predicting HER2-targeting benefit.

**Methods:**

Pearson’s Chi square test and logistic regression were used to compare pCR in the breast and nodes (ypT0/Tis ypN0). Kaplan–Meier estimates and Cox models were used to compare event-free and overall survival among subtypes.

**Results:**

Intrinsic subtypes were determined in 271 baseline core biopsy samples. The pCR rate among patients with HER2-enriched (HER2E) subtype was greater compared to other subtypes combined (120/197, 60.9% versus 19/74, 25.7%; *p *< 0.001). In multivariate analysis among patients receiving trastuzumab-containing regimens (with clinical factors and HER2E subtype as factors), HER2E subtype was most strongly associated with pCR [OR 8.41 (95% CI 2.52–28.1) *p *< 0.001]. Patients with HER2E tumors did not benefit more from dual HER2-targeted therapy versus trastuzumab. The pCR rate was higher among HER2E tumors versus other subtypes in both estrogen receptor-positive and -negative tumors (*p *≤ 0.001). Higher *ESR1* gene expression was associated with lower pCR rate. No association was observed between subtype and long-term outcomes.

**Conclusion:**

Patients with HER2E tumors were most likely to attain pCR versus other subtypes. HER2E subtype represents a favorable marker for predicting HER2-targeting benefit, particularly with trastuzumab-based therapies.

**Electronic supplementary material:**

The online version of this article (10.1007/s10549-019-05398-3) contains supplementary material, which is available to authorized users.

## Introduction

Survival is increased in the adjuvant and metastatic settings with human epidermal growth factor 2 (HER2)-targeted treatment in patients with HER2-positive breast cancer [1, 2]. A meta-analysis of neoadjuvant trials demonstrated that pathologic complete response (pCR) in HER2-positive tumors is most closely associated with favorable survival [3].

Randomized phase three trials have demonstrated heterogeneity in the pCR benefit of combining neoadjuvant lapatinib plus trastuzumab compared to trastuzumab alone when added to chemotherapy [4–6]. However, two meta-analyses of phase two and three trials demonstrated a statistically significant pCR benefit for the combination compared to trastuzumab alone [7, 8]. The National Surgical Adjuvant Breast and Bowel Project (NSABP) protocol B-41 was a three-arm randomized phase three trial that evaluated neoadjuvant trastuzumab, lapatinib, or both in patients receiving chemotherapy [5]. The pCR rate in breast and nodes (total pCR; ypN0/Tis ypN0) for each arm was 49.4%, 47.4%, and 60.2%, respectively (combination versus trastuzumab, *p *= 0.056). Although the combination was not superior, subgroup analysis suggested that patients with positive clinical nodal status at baseline achieved a significantly higher pCR rate with the combination compared to trastuzumab alone. The 5-year recurrence-free interval (RFI; time from surgery to local, regional, or distal recurrence) and overall survival (OS) did not show a significant benefit for either lapatinib or the combination versus trastuzumab [9]. However, exploratory analysis suggested that trastuzumab-containing regimens are superior to lapatinib alone in long-term outcomes (overall log-rank *p *= 0.05, RFI; *p *= 0.07, OS). Importantly, breast pCR was significantly associated with improved RFI (*p *= 0.0009) and OS (*p *= 0.0004).

Tumor intrinsic subtype, as measured by a 50-gene intrinsic subtype profile Prediction Analysis of Microarray 50 (PAM50), provides prognostic and predictive information beyond conventional determinants of hormone receptor (HR) and HER2 status in breast cancer [10]. The HER2-enriched (HER2E) breast cancer intrinsic subtype is predictive of attaining pCR in patients treated with lapatinib, trastuzumab, or the combination added to neoadjuvant chemotherapy [6, 11–13]. More precisely defining the use of HER2E to determine patients who benefit from single or dual HER2-targeted neoadjuvant therapy with or without chemotherapy is essential for clinical application.

The goal of this study is to determine whether intrinsic subtype by PAM50 can predict pCR and long-term outcomes in NSABP B-41. We hypothesized that the benefit of dual versus single HER2 targeting is limited to the HER2E subtype. This report adheres to REMARK criteria (Suppplementary material Table S6) [14].

## Materials and methods

### Study design and patients

NSABP B-41 was an open-label, three-arm phase three study performed from 2007 to 2011. Eligibility criteria and trial procedures have been previously reported [5]. Tumors were locally tested for HER2. For the current preplanned secondary analyses of B-41, any patient with an available baseline core biopsy sample, who had pCR ascertained, and who did not withdraw consent was eligible.

The primary objective of the current analyses was to determine the value of genomic subtypes as measured by PAM50 in predicting the chance of pCR among patients with HER2-positive tumors receiving neoadjuvant HER2-targeting regimens. Pathologic complete response was defined as the absence of any invasive component in the resected breast specimen and absence of cancer on hematoxylin and eosin (H&E) evaluation of all resected lymph nodes following completion of neoadjuvant therapy (ypT0/Tis ypN0) [3]. Secondary objectives included comparing patients with HER2E tumors to those with other subtypes in long-term outcomes (event-free survival [EFS] and OS) and determining whether patients with HER2E tumors benefitted more from dual versus single HER2-targeting regimens versus those with other subtypes in pCR, EFS, and OS. Event-free survival was defined as time from randomization to first local, regional, or distant recurrence, second primary, or death from any cause. Overall survival was defined as the time from randomization to death from any cause. Since lapatinib-alone regimens were shown to be numerically inferior to trastuzumab-containing regimens in the literature [4, 5], trastuzumab-containing arms were analyzed in these secondary analyses.

The clinical data are housed at the NSABP Biostatistical Center in Pittsburgh Pennsylvania. Since the data were anonymized to Georgetown investigators, a waiver from the Georgetown institutional review board was obtained to proceed with the study.

### Procedures

For each patient, serial 10 μm sections were cut from selected tissue blocks by the NSABP Department of Pathology. Unstained sections of formalin-fixed paraffin-embedded (FFPE) tumor samples and associated H&E stained slides were sent to the Genomic and Epigenomics Shared Resource (GESR) at Georgetown University Medical Center (GUMC) blinded to all clinical data. These samples underwent pathological examination to confirm diagnosis and identify malignant tissue in the GUMC Histopathology and Tissue Shared Resource (HTSR) to guide subsequent RNA isolation. Areas with tumor were microdissected from those slides using the H&E slides as templates. Total RNA was extracted from the microdissected tissues after deparaffinization using the Roche High Pure FFPET RNA Isolation Kit (Roche Molecular Systems, Pleasanton, CA) per manufacturer’s instructions. The RNA quantity was estimated with ultraviolet–visible spectrophotometry using the NanoDrop 1000 spectrophotometer (Thermo Fisher Scientific, Waltham, MA) to ensure sample purity (optical density 260/280 nm ratio 1.7–2.5). To assess RNA quality, samples were analyzed using the Agilent RNA 6000 Nano Kit and Agilent 2100 Bioanalyzer (Agilent, Santa Clara, CA). Degree of RNA integrity was assessed using the smear analysis function in the Agilent 2100 Expert Software to measure the percentage of RNA molecules > 300 bp. Final RNA concentration (12.5 ng/μL) was normalized across all samples before input.

From each sample, 150 ng of RNA was hybridized to the 72-plex human PAM50-research use only (PAM50-RUO) CodeSet (Prosigna™) and processed on the nCounter Sprint Profiler (NanoString Technologies, Seattle, WA) according to manufacturer protocols. The system uses sequence-specific probes that hybridize directly to the mRNA in solution: a reporter probe, which carries a target-specific, four-color, six-position fluorescent barcode, and a capture probe, which allows the complex to be immobilized for data collection. The PAM50 assay (using the PAM50-RUO CodeSet) simultaneously measures the expression levels of 50 target sequences, including eight endogenous invariant mRNA targets, six positive quality control targets, and eight negative quality control targets consisting of probes with no sequence homology to human RNA (Supplementary material Table S7) [15].

### Statistical methods

The Pearson Chi square test was used to compare treatment and stratification factors between patients who had PAM50 subtype determined from a core biopsy sample to the remaining B-41 patients. For the primary analysis, the Pearson’s Chi square test with continuity correction was used to test whether patients with HER2E tumors achieved a higher pCR rate than patients with other subtyped tumors. The Cochran-Mantel–Haenszel test was also performed with treatment as the stratification factor. Logistic regression models were used to test whether tumor subtype (HER2E versus others) is predictive of pCR in patients on trastuzumab-containing regimens with adjustment for clinical factors of age (≥ 50 versus  < 50 years), HR status (positive versus negative), clinical nodal status (positive versus negative), clinical tumor size (≥ 4.1 cm versus 2–4 cm), and treatment (trastuzumab plus lapatinib versus trastuzumab alone). Interaction between treatment and subtype was tested in the multivariate logistic regression model. The Breslow-Day test was also used to analyze whether patients with HER2E tumors benefitted more from dual-targeted therapy versus trastuzumab alone in pCR.

In the secondary analyses of EFS and OS, the log-rank test was used to test whether patients with HER2E tumors achieved better EFS or OS than patients with other subtyped tumors. Cox proportional hazards models were used to test whether tumor subtype (HER2E versus others) was independently predictive of EFS or OS with adjustment for treatment and HR status (positive/negative). Separate Cox models, based on data from patients receiving trastuzumab-based regimens, were used to test whether an interaction exists between tumor subtype (HER2E versus others) and dual targeting (trastuzumab plus lapatinib versus trastuzumab), and an interaction between the expression level of *ERBB2*, *ESR1*, and dual targeting. To study the prognostic utility of individual genes on pCR in patients receiving trastuzumab-containing regimens, univariate logistic regression models were used with the Benjamini–Hochberg procedure to control the false discovery rate (FDR) at 0.1 [16]. The Holm’s step-down procedure was used to identify gene signatures significantly prognostic for pCR with a familywise error rate (FWER) controlled at 0.05 [17]. Exploratory multivariate logistic regression models were used to predict pCR with clinical factors, the selected individual genes, and tumor subtype combined. Model selection was done via likelihood ratio test between the nested model and a sub-model, and the Akaike information criterion between non-nested models [18].

## Results

**Fig. 1:**
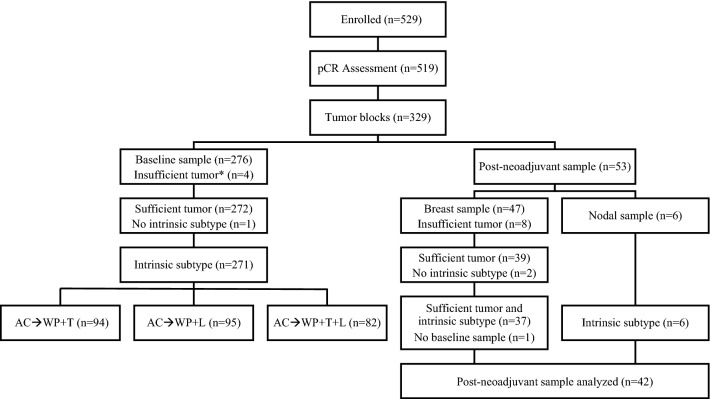
NSABP B-41 patient sample flowchart. *Samples either did not have tumor or had < 10% tumor cells in the available sections and were not assayed. *AC* doxorubicin and cyclophosphamide, *L* lapatinib, *pCR* pathologic complete response, *T* trastuzumab, *WP* weekly paclitaxel

A total of 329 tissue samples from 276 patients enrolled in B-41 were available, including 276 baseline samples from core biopsy prior to neoadjuvant regimens and 53 tissue samples (47 breast tissue and 6 lymph node) obtained at surgery after neoadjuvant treatment (Fig. 1). Twelve samples, four from core biopsy and eight breast tissue samples from surgery, either did not have tumor or had < 10% tumor cells in the available sections and were not assayed. Among the remaining baseline 272 core biopsy samples, intrinsic subtype was successfully determined in 271. Subsequent analyses are based on data from these patients. Comparison of baseline characteristics between the patients included and not included in the analysis showed no differences except for a higher proportion of estrogen receptor (ER)-negative cases in the included cohort (*p *= 0.01; Table 1).

**Table 1:** Baseline characteristics of patients included and excluded in the current analysis

Category	Characteristic	Included (*N *= 271), *N* (%)	Excluded (*N *= 258), *N* (%)	*p* value^a^
Treatment	AC → WP + T	94 (35)	87 (34)	0.37
	AC → WP + L	95 (35)	79 (31)	
	AC → WP + TL	82 (30)	92 (36)	
Clinical nodal status	N0	130 (48)	127 (49)	0.52
	N1+	141 (52)	131 (51)	
Age	< 50 years	135 (50)	142 (55)	0.23
	≥ 50 years	136 (50)	116 (45)	
Clinical tumor size	2.0–4.0 cm	136 (50)	135 (52)	0.62
	4.1 + cm	135 (50)	123 (48)	
Hormone receptor status	Negative	116 (43)	82 (32)	0.01
	Positive	155 (57)	176 (68)	

*AC* doxorubicin and cyclophosphamide, *L* lapatinib, *T* trastuzumab, *WP* weekly paclitaxel

^a^Pearson Chi square test

### pCR by intrinsic subtype

**Fig. 2:**
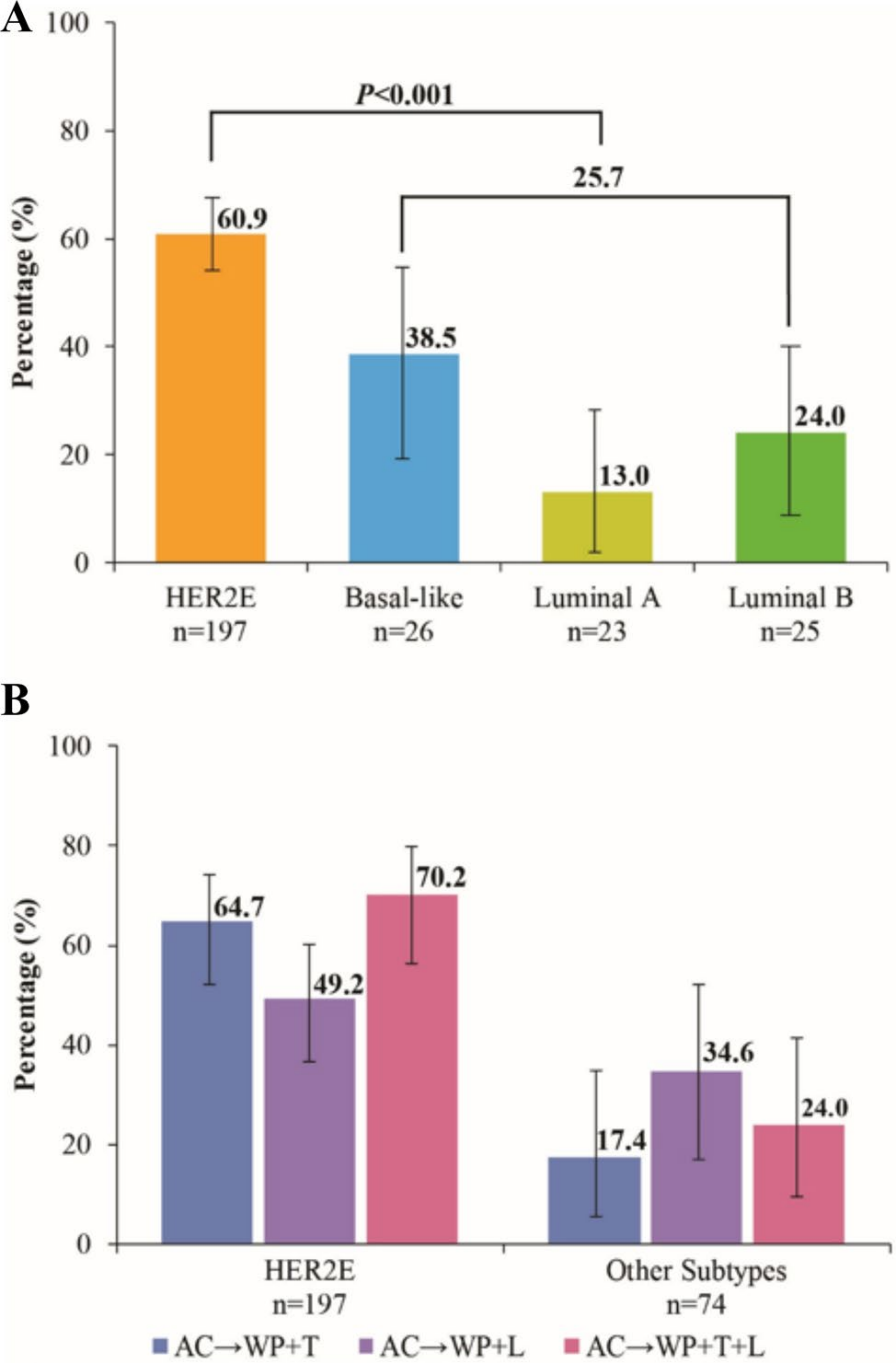
Total pCR (ypT0/Tis ypN0) by core biopsy intrinsic subtype. (**A**) Total pCR between HER2E and other subtypes and (**B**) total pCR by treatment and intrinsic subtype. Vertical bars represent 95% confidence intervals. *AC* doxorubicin and cyclophosphamide; *HER2E* HER2-enriched, *L* lapatinib, *T* trastuzumab, *WP* weekly paclitaxel

In the core biopsy intrinsic subtype analysis, pCR was achieved in 120 of 197 patients (60.9%, 95% CI 53.7–67.3%) with HER2E, 10 of 26 (38.5%, 95% CI 20.4–56.3%) with basal-like, 3 of 23 (13.0%, 95% CI 3.3–29.7%) with luminal A, and 6 of 25 (24.0%, 95% CI 9.8–41.7%) with luminal B subtype (Fig. 2a). The pCR rate among patients with HER2E subtype was statistically significantly greater compared to other subtypes combined [60.9% vs. 25.7% (95% CI 16.4–36.0%); *p *< 0.001].

All three HER2-targeted neoadjuvant regimens produced higher pCR rates in the HER2E population as compared to other intrinsic subtypes (Table 2; Fig. 2B). The odds ratio (OR) for pCR associated with HER2E (versus other subtypes) among patients treated with trastuzumab was 8.7 (95% CI 2.7–28.5). The corresponding OR for pCR among patients treated with trastuzumab plus lapatinib was 7.5 (95% CI 2.5–21.9).

**Table 2:** Pathologic complete response by treatment arm and intrinsic subtype (HER2E vs. others)

Treatment arm	Intrinsic subtype	N	pCR, N (%)	95% CI (%)	*p* value
AC → WP + T	HER2E	71	46 (64.7)	52.5–74.6	< 0.001
	Others^a^	23	4 (17.4)	5.4–35.0	
AC → WP + L	HER2E	69	34 (49.2)	37.1–60.4	0.29
	Others^a^	26	9 (34.6)	17.5–52.5	
AC → WP + T + L	HER2E	57	40 (70.2)	56.5–80.3	< 0.001
	Others^a^	25	6 (24.0)	9.8–41.7	

*AC* doxorubicin and cyclophosphamide, *HER2E* human epidermal growth factor receptor 2-enriched, *L* lapatinib, *pCR* pathologic complete response, *T* trastuzumab

^a^Includes luminal A, luminal B, and basal-like

Among patients treated with trastuzumab-containing neoadjuvant regimens, HER2E subtype was statistically significantly associated with higher pCR rate (Cochran-Mantel–Haenszel test *p *< 0.0001). In multivariate logistic regression analysis for pCR among patients receiving trastuzumab-containing regimens (*n *= 176) that included clinical factors and HER2E subtype as factors, HER2E subtype was strongly associated with pCR (OR 8.41 [95% CI 2.52–28.1] *p *< 0.001; Table 3). The test for interaction between dual targeting and HER2E subtype was not statistically significant (*p *= 0.94). The *p* value for the Breslow-Day test on homogeneity of the ORs is 0.85.

**Table 3:** Multivariate logistic regression analysis of total pCR among patients treated with trastuzumab or trastuzumab plus lapatinib (*N *= 176)

Factors	Odds ratio (95% CI)	*p* value^a^
Age (≥ 50 vs. < 50 years)	0.77 (0.39–1.52)	0.45
Hormone receptor status (positive vs. negative)	0.74 (0.37–1.50)	0.41
Clinical nodal status (positive vs. negative)	0.48 (0.24–0.98)	0.04
Tumor size (≥ 4.1 vs. 2–4 cm)	1.28 (0.64–2.55)	0.48
HER2E subtype	8.41 (2.52–28.1)	< 0.001
Dual targeting in HER2E tumors	1.29 (0.60–2.77)	0.67
Dual targeting in other subtypes	1.37 (0.33–5.74)	0.52

*HER2E* HER2-enriched, *pCR* pathologic complete response

^a^Wald test

**Fig. 3:**
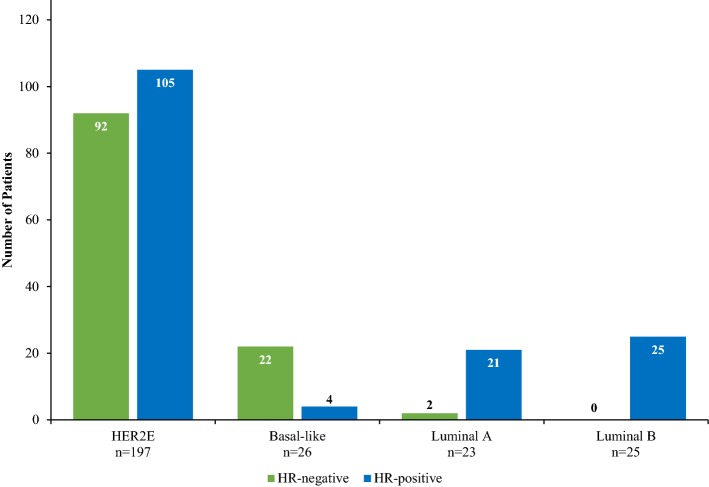
HR status according to intrinsic subtype. HR-positive indicates estrogen receptor or progesterone receptor positive. HR-negative indicates estrogen receptor and progesterone receptor negative. *HER2E* HER2-enriched, *HR* hormone receptor

The HR status of tumors according to intrinsic subtype is shown in Fig. 3. Of HER2E tumors, 105 (53.3%) were HR-positive and 100 (50.8%) were ER-positive. According to ER status, the proportions of patients achieving pCR were 69.1% (67/97, 95% CI 58.8–77.2%) for HER2E and ER-negative tumors, 53.0% (53/100, 95% CI 42.8–62.2%) for HER2E and ER-positive tumors, 29.2% (7/24, 95% CI 13.0–47.6%) for ER- negative tumors of other subtypes combined, and 24.0% (12/50, 95% CI 13.3–36.4%) for ER-positive tumors of other subtypes combined. The pCR rate among HER2E tumors versus other subtypes was statistically significantly greater in both ER-positive (*p *= 0.001) and ER-negative (*p *< 0.001) subgroups.

### pCR by gene expression analysis

**Fig. 4:**
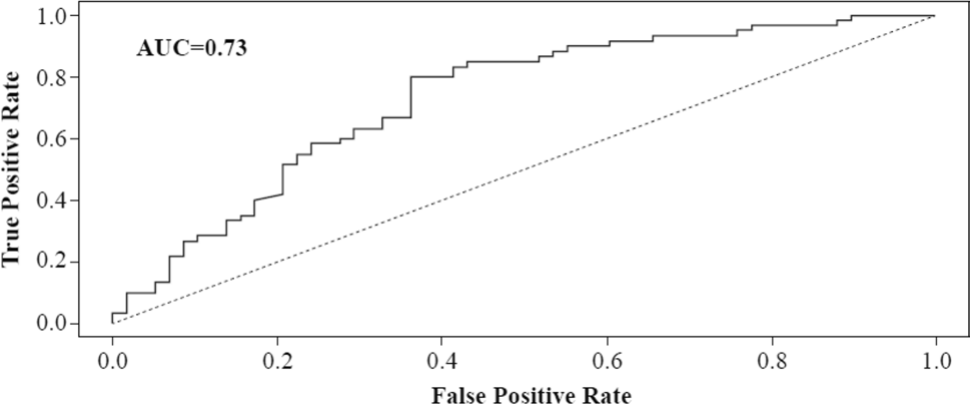
Receiver operating characteristics curve for predicting total pCR among patients in the testing set (*n *= 58) based on the logistic regression model developed from the training set (*n *= 118). Involved predictors were clinical nodal status, *ESR1* gene expression, and subtype (HER2E vs. others). *AUC* area under the curve

In patients on trastuzumab-containing regimens, 14 among 50 genes on the PAM50 panel were associated with pCR with FDR controlled at 0.1. These genes were *GRB7*, *ERBB2*, *PGR*, *MYC*, *ESR1*, *MAPT*, *BCL2*, *TMEM45B*, *CDC6*, *SLC39A6*, *CEP55*, *KIF2C*, *RRM2*, and *PHGDH* (Table 4). Among these 14 genes, *GRB7* (OR 1.58, adjusted *p *= 0.003), *ERBB2* (OR 1.6, adjusted *p *= 0.007), *PGR* (OR 0.71, adjusted *p *= 0.025), and *MYC* (OR 0.57, adjusted *p *= 0.026) were predictive of pCR with FWER controlled at 0.05. Correlations among expression levels of these four genes were present with the Pearson correlation coefficients varying from 0.3 to 0.87. Multivariable logistic regression models were used to combine clinical factors, such as ER status and nodal status; expression levels of *GRB7*, *ERBB2*, *PGR*, *MYC*, and *ESR1*; and the intrinsic subtype (HER2E versus others). Based on the Akaike information criterion, *ESR1* provided more prognostic power than ER status, and the intrinsic subtype was superior in prognostic value to *ERBB2* or *GRB7*. In the final multivariable logistic regression model, statistically significant predictors of pCR were clinical node positivity (OR 0.50, 95% CI 0.25–0.98, *p *= 0.04), *ESR1* expression (OR 0.86, 95% CI 0.74–0.99, *p *= 0.04, for doubling expression level), and HER2E subtype (OR 6.82, 95% CI 2.98–15.5, *p *< 0.0001). To assess the prognostic utility of nodal status, *ESR1* expression, and molecular subtype (HER2E versus others), patients on trastuzumab-based regimens were randomly split into a training set of 118 patients and a testing set of 58 patients. A multivariate logistic regression model was then fit to predict pCR with these three factors using data from the training set, and the resulting linear combination from the fitted logistic model was applied to data from patients in the testing set as an index for pCR. The resulting area under the receiver operating characteristic curve is 0.73, indicating that molecular subtype and *ESR1* expression are strong and informative prognostic factors for pCR in patients receiving HER2-targeted therapy (Fig. 4).

**Table 4:** Individual gene signatures prognostic for total pCR among patients treated with trastuzumab or trastuzumab plus lapatinib (*n *= 176) with false discovery rate controlled at 0.1

Gene signatures	OR^a^	*p* value^b^	Adjusted *p* value^c^
*GRB7*	1.58	6.8E−5	0.003
*ERBB2*	1.60	1.5E−4	0.007
*PGR*	0.71	5.2E−4	0.025
*MYC*	0.57	5.5E−4	0.026
*ESR1*	0.80	0.001	0.051
*MAPT*	0.70	0.001	0.065
*BCL2*	0.67	0.004	0.19
*TMEM45B*	1.41	0.006	0.25
*CDC6*	1.40	0.008	0.32
*SLC39A6*	0.71	0.008	0.34
*CEP55*	1.91	0.01	0.40
*KIF2C*	1.60	0.02	0.63
*RRM2*	1.64	0.02	0.68
*PHGDH*	1.36	0.02	0.68

*pCR* pathologic complete response

^a^Univariate logistic regression models

^b^Univariate logistic regression models with total pCR as the outcome variable

^c^Holm’s step-down procedure to adjust for multiple testings

### Residual disease

Thirty-six patients with breast tissue samples and six patients with axillary node samples (*n *= 42) were analyzed for agreement in intrinsic subtype between core biopsy samples and residual breast tissues or lymph nodes at surgery (Table 5). Moderate agreement was observed [weighted Kappa statistic = 0.49 (95% CI 0.30–0.67)].

**Table 5:** Correlation of intrinsic subtype between core biopsy and primary surgery^a^

Core biopsy subtype	Surgical sample subtype, *N*				
	HER2E	Basal-like	Luminal A	Luminal B	Total
HER2E	12	1	6	1	20
Basal-like	0	4	0	0	4
Luminal A	1	0	6	1	8
Luminal B	1	0	8	1	10
Total	14	5	20	3	42

^a^Weighted Kappa statistic = 0.49 (95% CI 0.30–0.67)

*HER2E* HER2-enriched

### Long-term outcomes

**Fig. 5:**
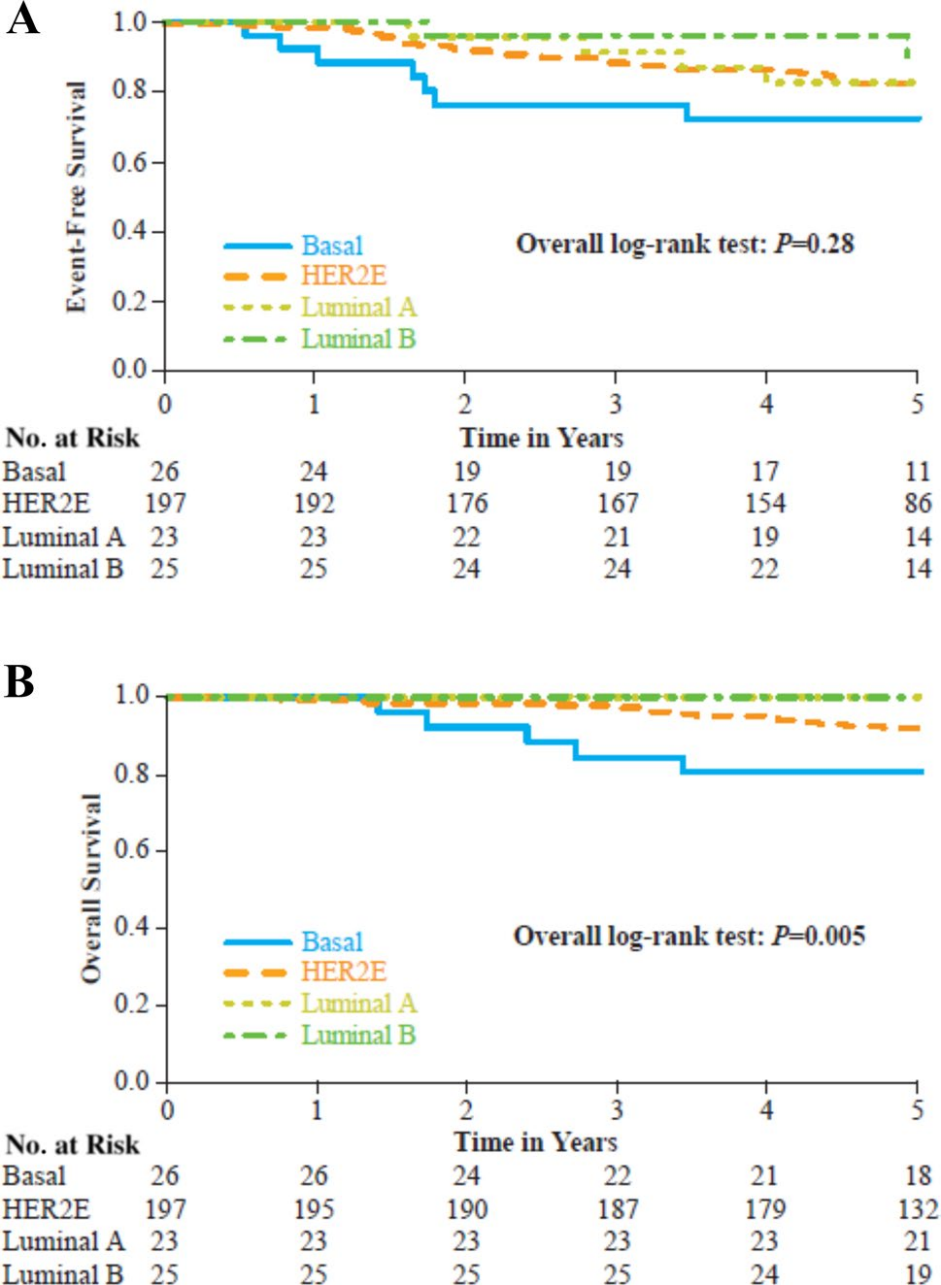
Long-term outcomes. (**A**) Event-free survival and (**B**) overall survival. *HER2E* HER2-enriched

Among the 271 patients with core biopsy intrinsic subtype analysis, median follow-up was 5.1 years. A total of 48 EFS events occurred. Five-year EFS rates were 82.3% (95% CI 75.9–87.1%) and 81.6% (95% CI 70.2–88.9%) for patients with HER2E and other subtypes, respectively. No statistically significant difference in EFS was observed between HER2E and other subtypes [hazard ratio = 0.69 (95% CI 0.26–1.81), log-rank *p *= 0.89). The overall log-rank *p* value for EFS among all subtypes was 0.28 (Fig. 5a). The Kaplan–Meier estimates of 5-year EFS rates by pCR and subtype are shown for patients who received trastuzumab-based regimens in Table 6. Among patients who did not achieve pCR, those with HER2E subtype had numerically worse prognosis than those with other subtypes. Conversely, among patients who achieved pCR, those with HER2E subtype had numerically better prognosis than those with other subtypes.

**Table 6:** Kaplan–Meier estimates of 5-year EFS by pCR and PAM50 subtype in patients treated with trastuzumab-containing neoadjuvant regimens

pCR status	PAM50 subtype	No. of events/No. of patients	5-year EFS (%) (95% CI)
No pCR	HERE	12/42	73.0 (56.6, 84.1)
	Other	7/38	81.5 (65.1, 90.7)
pCR	HER2E	8/86	90.1 (81.2, 94.9)
	Other	2/10	80.0 (40.9, 94.6)

*EFS* event-free survival, *HER2E* HER2-enriched, *PAM50* prediction analysis of microarray 50, *pCR* pathologic complete response in breast and nodes (ypN0/Tis ypN0)

A total of 21 deaths occurred. Five-year OS rates were 92.1% (95% CI 87.1–95.1%) and 93.2% (95% CI 84.5–97.1%) for patients with HER2E and other subtypes, respectively. No statistically significant difference in OS was observed between HER2E and other subtypes [hazard ratio = 0.79 (95% CI 0.41–1.52) *p *= 0.98]. However, the overall log-rank *p* value for OS among all subtypes was 0.005 (Fig. 5b), with shortened OS in the basal-like subgroup (5-year OS rate 80.8%; *p *= 0.01 vs. HER2E subtype).

## Discussion

This analysis of NSABP B-41 outcomes according to intrinsic subtypes as determined by PAM50 demonstrated that the HER2E subtype was statistically significantly associated with achieving pCR compared to other intrinsic subtypes in HER2-positive patients receiving trastuzumab-containing neoadjuvant regimens. This association was maintained regardless of ER status.

Selecting patients for HER2-targeted therapy remains a priority to ensure that those likely to benefit receive the most effective treatment and to avoid unnecessary costs and potential adverse events in patients unlikely to benefit. While current standard-of-care methods of determining HER2 positivity accurately identify a subset of patients who benefit from neoadjuvant HER2-targeted therapy, a substantial portion of HER2-positive patients fail to attain pCR [4, 19]. Intrinsic subtype provides valuable information about the sensitivity of tumors to a variety of currently available agents including HER2-targeted agents, cytotoxic chemotherapy, and cyclin-dependent kinase inhibitors [10, 20]. Genomic analysis is now being evaluated for its ability to improve identification of patients who will respond to neoadjuvant HER2-targeted therapy [6, 11–13, 21]. Fumagalli, et al. demonstrated that among patients treated with neoadjuvant trastuzumab, lapatinib, or the combination with chemotherapy in NeoALTTO, the HER2E subtype was associated with a pCR rate of 52% compared to 17–38% across other subtypes [13]. Similarly, Carey, et al. demonstrated a higher pCR rate among HER2E tumors compared to other subtypes in patients receiving neoadjuvant trastuzumab with or without lapatinib plus chemotherapy in CALGB 40601 [6]. Among all treatment arms, pCR rates were 66%, 34%, and 34% for HER2E, luminal A, and luminal B subtypes, respectively. The current analysis of NSABP B-41 adds to this body of literature by demonstrating the benefit of intrinsic subtype as measured by PAM50 in predicting the benefit of HER2-targeting therapy. Patients with HER2E tumors were statistically significantly more likely to benefit from neoadjuvant HER2-targeted therapy added to chemotherapy than patients with basal-like, luminal A, or luminal B subtype tumors. The pCR rate in the HER2E subgroup was 61%, consistent with NeoALTTO and CALGB40601. Conversely, pCR rates among patients in our analysis with other subtypes ranged from 13 to 38%, consistent with pCR achieved with chemotherapy alone [22]. As such, whether HER2-targeted therapy offers benefit beyond chemotherapy or endocrine therapy in patients with HER2-positive disease and non-HER2E subtype requires investigation in future prospective clinical trials.

In a different approach, neoadjuvant trastuzumab plus lapatinib was compared to trastuzumab alone without chemotherapy in the single-arm PAMELA study [21]. Intrinsic subtype analysis also demonstrated an increased breast pCR rate among patients with HER2E tumors (41%) compared to other subtypes (10%). Although the breast pCR rate was lower than observed with regimens incorporating chemotherapy, these data provide a potential signal for eliminating chemotherapy in a subset of patients selected based on intrinsic subtype. A combined genomic marker incorporating HER2E and ERBB2-high mRNA was associated with increased pCR compared to other subtype and ERBB2-low tumors when applied to data from 5 trials, including PAMELA, that evaluated single versus dual HER2-targeted therapy without chemotherapy in localized or advanced breast cancer. [23].

Prior data have demonstrated a lower pCR rate in response to neoadjuvant trastuzumab, lapatinib, or the combination among patients with HR-positive as compared to HR-negative disease [3, 4, 24]. In our analysis, pCR rates were 69.1% for HER2E/ER-negative tumors and 53.0% for HER2E/ER-positive tumors, suggesting that pCR rate remains decreased among HR-positive tumors within the HER2E subtype. However, the pCR rate among HER2E tumors versus other subtypes was statistically significant in both ER-positive (*p *= 0.001) and ER-negative (*p *< 0.001) tumors, demonstrating a benefit in all patients with HER2E tumors regardless of HR status. Also, a study evaluating lapatinib and letrozole in first-line HR-positive breast cancer showed that patients with HER2-negative/HER2E tumors benefited from lapatinib therapy [25]. These data suggest that there is value in intrinsic subtyping of ER-positive tumors.

Individual gene expressions from the PAM50 panel associated with pCR were *ERBB2* and *ESR1*: higher *ERBB2* level and lower *ESR1* level were associated with increasing pCR rate. This finding is consistent with RNA sequencing data from CALGB 40601 [6] and NeoALLTO [13].

In the NSABP B-41 primary analysis, dual HER2 targeting with trastuzumab plus lapatinib did not statistically significantly increase the pCR rates in the breast or breast plus nodes as compared to trastuzumab alone [5]. Results of the current analysis are consistent with this finding. While all three HER2-targeted neoadjuvant regimens produced higher pCR rates in the HER2E population as compared to other intrinsic subtypes, patients with HER2E tumors did not benefit more from dual-targeted therapy versus trastuzumab alone. As has been reported, the trastuzumab arm experienced a higher pCR rate than expected, which may have affected the ability of NSABP B-41 to detect a statistically significant difference between dual-targeted therapy and trastuzumab alone [5]. Despite the lack of lapatinib combination therapy benefit, the PAM50 analysis demonstrated an ability to identify patients more likely to benefit from both trastuzumab-based regimens evaluated.

Our study did not identify an EFS benefit among patients with HER2E tumors versus other subtypes. Interestingly, Fernandez-Martinez, et al. preliminarily reported an EFS benefit among patients with HER2E tumors who received dual-HER2-targeted trastuzumab plus lapatinb plus chemotherapy versus trastuzumab plus chemotherapy in CALGB40601, but not among patients with other subtypes. [26].

There is also evidence from other HER2 targeted therapy trials that HER2E subtype predicts pCR. Initial evidence has demonstrated a breast and pCR benefit, respectively, for dual HER2 targeting with trastuzumab plus pertuzumab, including a differential benefit in patients with HER2E tumors [18, 27].

The 42 matched paired pre- and post-treatment samples revealed at surgery a result of HER2E subtype in 12 (60.0%) and luminal A subtype in 6 (30.0%). This pattern differed from that observed in CALGB 40601, where a majority of HERE subtype tumors demonstrated post-treatment alterations to luminal A subtype [6 of 9 (66.7%)] after HER2-targeted therapy plus chemotherapy [6]. It is possible that these differences are due to tumor heterogeneity, but post-treatment subtype alterations have important implications for treatment of residual disease.

Strengths of the current analysis include use of a large dataset with biomarkers from a randomized controlled trial and use of stringent pCR criteria (ypT0/Tis ypN0) consistent with current standards of reporting. Limitations include the limited number of patients with tumor blocks resulting in partial inclusion of study participants from the parent study and the low number of event-free and overall survival events. Meta-analyses with other similar studies will provide valuable additional information about these long-term outcomes in patients with early breast cancer.

Results of the current analysis indicate that the HER2E subtype may represent a favorable marker for predicting benefit from HER2-targeted agents, particularly trastuzumab-based therapies. Available evidence suggests a potential role for intrinsic subtype in identifying appropriate patients for neoadjuvant HER2-targeted therapy. These results also underscore the need to elucidate the role of intrinsic subtype for treatment selection in patients without HER2E tumors and identify appropriate treatment for these patients who experience low pCR rates. Prospective trials of neoadjuvant anti-HER2 therapy with randomization according to intrinsic subtype are now warranted in order to optimize treatment for patients without HER2E subtype tumors. Future directions include an ongoing meta-analysis of intrinsic subtype data to thoroughly evaluate the benefit of pCR on event-free and overall survival, and the use of proteomic analysis in combination with intrinsic subtype to further refine patient selection for specific therapies [28].

## Electronic supplementary material

Below is the link to the electronic supplementary material.

Supplementary material 1 (DOCX 27 kb)

## Data Availability

The datasets during and/or analyzed during the current study are available from the corresponding author on reasonable request.
