# Supplementary material for: Pathologic complete response and outcomes by intrinsic subtypes in NSABP B-41, a randomized neoadjuvant trial of chemotherapy with trastuzumab, lapatinib, or the combination
Source: Breast Cancer Res Treat. 2019 Aug 19;178(2):389–99. doi: 10.1007/s10549-019-05398-3 (PMC6797698; doi:10.1007/s10549-019-05398-3)
Supplement: Supplementary file 1 — Supplementary material 1 (DOCX 27 kb) [file 10549_2019_5398_MOESM1_ESM.docx]

**ELECTRONIC SUPPLEMENTAL INFORMATION**

*Breast Cancer Research and Treatment*

**Pathologic complete response and outcomes by intrinsic subtypes in NSABP B-41, a randomized neoadjuvant trial of chemotherapy with trastuzumab, lapatinib, or the combination**

Swain SM^^[[1]](#footnote-1)^^ (Georgetown Lombardi Comprehensive Cancer Center, e-mail: [sandra.swain@georgetown.edu](mailto:sandra.swain@georgetown.edu)), Tang G, Lucas PC, Robidoux A, Goerlitz D, Harris BT, Bandos H, Geyer CE, Rastogi P, Mamounas EP, Wolmark N

**TABLE OF CONTENTS**

Table S6. REMARK Checklist 2

Table S7. nCounter PAM50 Gene List 3

**Table S6. REMARK Checklist^^[[2]](#footnote-2)^^**

| **Item to be reported** | | **Page no.** |
| --- | --- | --- |
| **Introduction** | |  |
| 1 | State the marker examined, the study objectives, and any pre-specified hypotheses. | 3,4 |
| **Materials and Methods** | |  |
| *Patients* | |  |
| 2 | Describe the characteristics (e.g., disease stage or co-morbidities) of the study patients, including their source and inclusion and exclusion criteria. | 3 |
| 3 | Describe treatments received and how chosen (e.g., randomized or rule-based). | 3 |
| *Specimen characteristics* | |  |
| 4 | Describe type of biological material used (including control samples) and methods of preservation and storage. | 4 |
| *Assay methods* | |  |
| 5 | Specify the assay method used and provide (or reference) a detailed protocol, including specific reagents or kits used, quality control procedures, reproducibility assessments, quantitation methods, and scoring and reporting protocols. Specify whether and how assays were performed blinded to the study endpoint. | 4, 5 |
| *Study design* | |  |
| 6 | State the method of case selection, including whether prospective or retrospective and whether stratification or matching (e.g., by stage of disease or age) was used. Specify the time period from which cases were taken, the end of the follow-up period, and the median follow-up time. | 3, 7 |
| 7 | Precisely define all clinical endpoints examined. | 4 |
| 8 | List all candidate variables initially examined or considered for inclusion in models. | 5 |
| 9 | Give rationale for sample size; if the study was designed to detect a specified effect size, give the target power and effect size. | 3, 5 |
| *Statistical analysis methods* | |  |
| 10 | Specify all statistical methods, including details of any variable selection procedures and other model-building issues, how model assumptions were verified, and how missing data were handled. | 5 |
| 11 | Clarify how marker values were handled in the analyses; if relevant, describe methods used for cutpoint determination. | 5 |
| **Results** | |  |
| *Data* | |  |
| 12 | Describe the flow of patients through the study, including the number of patients included in each stage of the analysis (a diagram may be helpful) and reasons for dropout. Specifically, both overall and for each subgroup extensively examined report the numbers of patients and the number of events. | 5, 6, Fig. 1 |
| 13 | Report distributions of basic demographic characteristics (at least age and sex), standard (disease-specific) prognostic variables, and tumor marker, including numbers of missing values. | 5, 6, 15 |
| *Analysis and presentation* | |  |
| 14 | Show the relation of the marker to standard prognostic variables. | 6, 16, Fig. 3 |
| 15 | Present univariable analyses showing the relation between the marker and outcome, with the estimated effect (e.g., hazard ratio and survival probability). Preferably provide similar analyses for all other variables being analyzed. For the effect of a tumor marker on a time-to-event outcome, a Kaplan-Meier plot is recommended. | 6, 7, Fig. 5 |
| 16 | For key multivariable analyses, report estimated effects (e.g., hazard ratio) with confidence intervals for the marker and, at least for the final model, all other variables in the model. | 6, 7, 18 |
| 17 | Among reported results, provide estimated effects with confidence intervals from an analysis in which the marker and standard prognostic variables are included, regardless of their statistical significance. | 17 |
| 18 | If done, report results of further investigations, such as checking assumptions, sensitivity analyses, and internal validation. | 7 |
| **Discussion** | |  |
| 19 | Interpret the results in the context of the pre-specified hypotheses and other relevant studies; include a discussion of limitations of the study. | 7, 8, 9 |
| 20 | Discuss implications for future research and clinical value. | 7, 9, 10 |

**Table S7. nCounter PAM50 Gene List**

| Official Symbol | Accession | Official Full Name |
| --- | --- | --- |
| *UBE2T* | NM_014176.1 | Homo sapiens ubiquitin-conjugating enzyme E2T (putative) (UBE2T) |
| *PTTG1* | NM_004219.2 | Homo sapiens pituitary tumor-transforming 1 (PTTG1) |
| *PGR* | NM_000926.2 | Homo sapiens progesterone receptor (PGR) |
| *MKI67* | NM_002417.2 | Homo sapiens antigen identified by monoclonal antibody Ki-67 (MKI67) |
| *MIA* | NM_006533.1 | Homo sapiens melanoma inhibitory activity (MIA) |
| *MAPT* | NM_016835.3 | Homo sapiens microtubule-associated protein tau (MAPT) |
| *KRT17* | NM_000422.1 | Homo sapiens keratin 17 (KRT17) |
| *KRT14* | NM_000526.3 | Homo sapiens keratin 14 (epidermolysis bullosa simplex, Dowling-Meara, Koebner) (KRT14) |
| *KIF2C* | NM_006845.2 | Homo sapiens kinesin family member 2C (KIF2C) |
| *ESR1* | NM_000125.2 | Homo sapiens estrogen receptor 1 (ESR1) |
| *CCNE1* | NM_001238.1 | Homo sapiens cyclin E1 (CCNE1) |
| *CENPF* | NM_016343.3 | Homo sapiens centromere protein F, 350/400ka (mitosin) (CENPF) |
| *CEP55* | NM_018131.3 | Homo sapiens centrosomal protein 55kDa (CEP55) |
| *FGFR4* | NM_002011.3 | Homo sapiens fibroblast growth factor receptor 4 (FGFR4) |
| *MMP11* | NM_005940.3 | Homo sapiens matrix metallopeptidase 11 (stromelysin 3) (MMP11) |
| *SFRP1* | NM_003012.3 | Homo sapiens secreted frizzled-related protein 1 (SFRP1) |
| *TMEM45B* | NM_138788.3 | Homo sapiens transmembrane protein 45B (TMEM45B) |
| *TYMS* | NM_001071.1 | Homo sapiens thymidylate synthetase (TYMS) |
| *ERBB2* | NM_004448.2 | Homo sapiens v-erb-b2 erythroblastic leukemia viral oncogene homolog 2, neuro/glioblastoma derived oncogene homolog (avian) (ERBB2) |
| *CDCA1* | NM_145697.1 | Homo sapiens cell division cycle associated 1 (CDCA1) |
| *BCL2* | NM_000633.2 | Homo sapiens B-cell CLL/lymphoma 2 (BCL2), nuclear gene encoding mitochondrial protein |
| *CCNB1* | NM_031966.2 | Homo sapiens cyclin B1 (CCNB1) |
| *CDC20* | NM_001255.1 | Homo sapiens CDC20 cell division cycle 20 homolog (S. cerevisiae) (CDC20) |
| *NAT1* | NM_000662.4 | Homo sapiens N-acetyltransferase 1 (arylamine N-acetyltransferase) (NAT1) |
| *ORC6L* | NM_014321.2 | Homo sapiens origin recognition complex, subunit 6 like (yeast) (ORC6L) |
| *RRM2* | NM_001034.1 | Homo sapiens ribonucleotide reductase M2 polypeptide (RRM2) |
| *UBE2C* | NM_007019.2 | Homo sapiens ubiquitin-conjugating enzyme E2C (UBE2C) |
| *ACTR3B* | NM_001040135.1 | Homo sapiens ARP3 actin-related protein 3 homolog B (yeast) (ACTR3B) |
| *ANLN* | NM_018685.2 | Homo sapiens anillin, actin binding protein (scraps homolog, Drosophila) (ANLN) |
| *BAG1* | NM_004323.3 | Homo sapiens BCL2-associated athanogene (BAG1) |
| *BIRC5* | NM_001168.2 | Homo sapiens baculoviral IAP repeat-containing 5 (survivin) (BIRC5) |
| *BLVRA* | NM_000712.3 | Homo sapiens biliverdin reductase A (BLVRA) |
| *CDC6* | NM_001254.3 | Homo sapiens CDC6 cell division cycle 6 homolog (S. cerevisiae) (CDC6) |
| *CDH3* | NM_001793.3 | Homo sapiens cadherin 3, type 1, P-cadherin (placental) (CDH3) |
| *CXXC5* | NM_016463.5 | Homo sapiens CXXC finger 5 (CXXC5) |
| *EGFR* | NM_005228.3 | Homo sapiens epidermal growth factor receptor (erythroblastic leukemia viral (v-erb-b) oncogene homolog, avian) (EGFR) |
| *EXO1* | NM_006027.3 | Homo sapiens exonuclease 1 (EXO1) |
| *FOXA1* | NM_004496.2 | Homo sapiens forkhead box A1 (FOXA1) |
| *FOXC1* | NM_001453.1 | Homo sapiens forkhead box C1 (FOXC1) |
| *GPR160* | NM_014373.1 | Homo sapiens G protein-coupled receptor 160 (GPR160) |
| *GRB7* | NM_005310.2 | Homo sapiens growth factor receptor-bound protein 7 (GRB7) |
| *KNTC2* | NM_006101.1 | Homo sapiens kinetochore associated 2 (KNTC2) |
| *KRT5* | NM_000424.2 | Homo sapiens keratin 5 (epidermolysis bullosa simplex, Dowling-Meara/Kobner/Weber-Cockayne types) (KRT5) |
| *MDM2* | NM_006878.2 | Homo sapiens Mdm2, transformed 3T3 cell double minute 2, p53 binding protein (mouse) (MDM2) |
| *MELK* | NM_014791.2 | Homo sapiens maternal embryonic leucine zipper kinase (MELK) |
| *MLPH* | NM_024101.4 | Homo sapiens melanophilin (MLPH) |
| *MYBL2* | NM_002466.2 | Homo sapiens v-myb myeloblastosis viral oncogene homolog (avian)-like 2 (MYBL2) |
| *MYC* | NM_002467.3 | Homo sapiens v-myc myelocytomatosis viral oncogene homolog (avian) (MYC) |
| *PHGDH* | NM_006623.2 | Homo sapiens phosphoglycerate dehydrogenase (PHGDH) |
| *SLC39A6* | NM_012319.2 | Homo sapiens solute carrier family 39 (zinc transporter), member 6 (SLC39A6) |
| Reference Genes | | |
| *TFRC* | NM_003234.1 | Homo sapiens transferrin receptor (p90, CD71) (TFRC) |
| *ACTB* | NM_001101.2 | Homo sapiens actin, beta (ACTB) |
| *MRPL19* | NM_014763.3 | Homo sapiens mitochondrial ribosomal protein L19 (MRPL19) |
| *PSMC4* | NM_006503.2 | Homo sapiens proteasome (prosome, macropain) 26S subunit, ATPase, 4 (PSMC4) |
| *PUM1* | NM_001020658.1 | Homo sapiens pumilio RNA-binding family member 1 (PUM1) |
| *RPLP0* | NM_001002.3 | Homo sapiens ribosomal protein, large, P0 (RPLP0) |
| *SF3A1* | NM_005877.4 | Homo sapiens splicing factor 3a, subunit 1, 120kDa (SF3A1) |
| *GUSB* | NM_000181.1 | Homo sapiens glucuronidase, beta (GUSB) |

1. *Corresponding author* [↑](#footnote-ref-1)
2. McShane LM, Altman DG, Sauerbrei W, Taube SE, Gion M, Clark GM: Reporting recommendations for tumor marker prognostic studies (REMARK). *J Natl Cancer Inst* 2005; 97: 1180-1184. [↑](#footnote-ref-2)
